# Protective effect of dexmedetomidine against organ dysfunction in a two-hit model of hemorrhage/resuscitation and endotoxemia in rats

**DOI:** 10.1590/1414-431X20187905

**Published:** 2019-02-25

**Authors:** Yuanxu Jiang, Mingzhu Xia, Qiang Huang, Dengfeng Ding, Yali Li, Zhongjun Zhang, Xueping Zhang

**Affiliations:** 1 Department of Anesthesiology, Shenzhen People's Hospital, Shenzhen Anesthesiology Engineering Center, the Second Clinical Medical College, Jinan University, Shenzhen, China; 2 Hubei Community Health Service Center, Luohu Hospital group, Shenzhen, China

**Keywords:** Hemorrhagic shock, Resuscitation, Endotoxemia, Dexmedetomidine, Yohimbine

## Abstract

Dexmedetomidine (DEX), a selective agonist of α_2_-adrenergic receptors, has anti-inflammation properties and potential beneficial effects against trauma, shock, or infection. Therefore, this study aimed to investigate whether DEX might protect against multiple-organ dysfunction in a two-hit model of hemorrhage/resuscitation (HS) and subsequent endotoxemia. Eighty Wistar rats were randomized into four groups: NS (normal saline), HS/L (HS plus lipopolysaccharide), HS/L+D (HS/L plus dexmedetomidine), and HS/L+D+Y (HS/L+D plus yohimbine). Six hours after resuscitation, blood gas (PaO_2_) and serum alanine aminotransferase (ALT), aspartate aminotransferase (AST), blood urine nitrogen (BUN), creatinine (Cr), TNF-α, IL-β, IL-6, IL-8, IL-10, and nitric oxide (NO) were measured. The histopathology was assayed by staining. Malondialdehyde (MDA) and superoxide dismutase (SOD) levels and heme oxygenase-1 (HO-1) were assayed. The PaO_2_ levels in HS/L rats were lower whereas the ALT, AST, BUN, Cr, TNF-α, IL-β, IL-6, IL-8, IL-10, and NO levels were higher compared to the control group. The HS/L+D increased PaO_2_ and further increased IL-10 and decreased ALT, AST, BUN, Cr, TNF-α, IL-β, IL-6, IL-8, and NO levels of the HS/L groups. In addition, the MDA in the HS/L groups increased whereas SOD activity decreased compared to the control group. Moreover, the HO-1 expression levels were increased by DEX administration in lung, liver, and kidney tissues. Lungs, livers, and kidneys of the HS/L group displayed significant damage, but such damage was attenuated in the HS/L+D group. All of the above-mentioned effects of DEX were partly reversed by yohimbine. DEX reduced multiple organ injury caused by HS/L in rats, which may be mediated, at least in part, by α_2_-adrenergic receptors.

## Introduction

The current two-hit hypothesis suggests that trauma (hemorrhage/resuscitation) creates a systemic inflammatory environment that is more sensitive to bacterial toxins (1). Endotoxemia induces the production of various inflammatory cytokines such as tumor necrosis factor (TNF-α) and interleukin-1ß (IL-1β) as well as reactive oxygen species (ROS). These mediators alone, or through their interactions, lead to neutrophil accumulation, local inflammation, and secretion of secondary proinflammatory cytokines and chemokines that eventually trigger a systemic inflammatory response syndrome and multiple organ dysfunction (MOD) (2).

Hemorrhagic shock leads to impaired delivery of oxygen to organs, and the main management during the first hit is to maintain oxygen delivery to prevent hypoxia, ischemia, inflammation, and organ dysfunction. This is achieved with the use of fluids, vasopressors, and blood transfusion (3). If endotoxemia occurs, the standard approach also includes fluids and vasopressors in addition to respiratory and renal support (4). However, these approaches are ineffective at controlling the inflammatory response.

Dexmedetomidine (DEX), a selective agonist of α_2_-adrenergic receptors, has sedative, analgesic, and sympatholytic properties, and it has been used for analgesia, sedation, and anesthetic sparing. Apart from its sedative and analgesic properties, DEX has also demonstrated potential anti-inflammatory effects both *in vivo* and *in vitro* (5,6). In addition, DEX has been shown to protect organs against ischemia-reperfusion (I/R) injury and sepsis in experimental animal models (7–9).

However, it remains unknown whether DEX could exert multiple organ protective effects in a model of hemorrhage/resuscitation and subsequent endotoxemia. Therefore, this study aimed to examine the effects of DEX on lung injury, renal injury, and hepatic injury in a two-hit rat model of hemorrhage/resuscitation and endotoxemia.

## Material and Methods

### Animals

Eighty male Wistar rats (200-220 g, 6-8 weeks old) raised in a specific pathogen-free environment were provided by the Experimental Animal Center of Nanfang Medical University (China). The rats were housed in an environmentally controlled animal care facility where they were fed *ad libitum* and exposed to 12-h light/dark cycles. The protocol was approved by the Animal Care Committee of Nanfang Medical University and was carried out according to institutional guidelines for animal care and by the Guide for Care and Use of Laboratory Animals published by the United States National Institutes of Health.

### Two-hit model protocol

The two-hit model was induced as previously described (10). A PE-50 catheter was inserted into the left carotid artery to monitor blood pressure and into the right carotid artery to induce hemorrhage. Control animals underwent catheter insertion, but no blood was withdrawn or returned. Hemorrhage was induced by withdrawing blood into a heparinized syringe (0.025 mL/g weight) for 10 min to lower mean arterial pressure (MAP) to approximately 40-50 mmHg. MAP was constantly maintained by withdrawing or infusing blood, as needed, during a 60 min period. Then, resuscitation was performed by reinfusing the remaining withdrawn blood plus normal saline (2-fold the maximum blood volume drawn) over 10 min. Sixty minutes after resuscitation, *Escherichia coli* endotoxin lipopolysaccharide (LPS), 15 mg/kg, Serotype 0127:B8; Sigma-Aldrich, USA) was administered intravenously to induce endotoxemia.

### Animal preparation and experimental design

After having their skin shaved and disinfected with 70% ethanol, the rats were anesthetized with sodium pentobarbital (50 mg/kg, *ip* injection). The right carotid artery and left femoral vein of each rat were punctured with catheters (PE-50) for administration of drugs and measurements of hemodynamic parameters. A tracheostomy was performed, and a 14-gauge angiocatheter was inserted as a tracheostomy tube to keep the airway unobstructed. After tracheostomy, anesthesia was maintained by supplementary injections of pentobarbital (approximately 1-3 mg/kg per hour, *iv*), as required.

The animals were randomly assigned to four groups (n=20/group): normal saline control group (NS), hemorrhage/resuscitation plus LPS group (HS/L), HS/L+DEX group (HS/L+D), and HS/L+DEX+yohimbine group (HS/L+D+Y). Rats in the NS group were treated with 0.9% normal saline (5 mL/kg), without causing hemorrhage or endotoxemia. The HS/L+D group received DEX (1 μg/kg, intravenous infusion over 10 min), followed by a second DEX infusion (5.0 μg/kg per hour) beginning immediately after endotoxin administration and lasting until the end of the experiment (6 h). The DEX dose was determined based on previous studies (11). Rats in the HS/L+D+Y group received yohimbine (0.1 mg/kg, intravenous infusion over 5 min) immediately after the loading dose of DEX.

The arterial catheter was connected to a pressure transducer (P23ID, Statham, USA) for taking measurements of MAP and heart rate (HR), which were continuously recorded on a multichannel recorder (MacLab/4e, AD Instruments Pty Ltd., Australia). At the end of the experiment, 1.5 mL of blood was collected from each animal into a serum gel S/1.3 tube (Monovette, Sarstedt, Germany) from a catheter placed in the carotid artery and used to measure liver and renal function. Another sample of blood (1.0 mL) was collected for blood gas analysis. All rats were sacrificed by intravenous injection with high-dose pentobarbital (300 mg/kg). Samples of lung, liver, and kidney tissues were removed for further examination.

The study consisted of two parts. The first part was the survival study, for which 10 animals were used in each group. The second part was the biochemical and histological study, for which another 10 animals were used in each group. If animals died, new animals were modeled to maintain the number of rats at 10 for each group/experiment. Therefore, for the first part, 10 rats from each group were randomly selected and used to determine the survival rate at 72 h after LPS administration.

### Arterial oxygen tension (PaO_2_)

A 0.5-mL blood sample was collected from the right common carotid artery of each rat 6 h after LPS administration, and PaO_2_ was immediately measured with a blood gas analyzer (Stat Profile pHOx, Nova Biomedical Corporation, USA).

### Biochemical indicators of organ injury

A 2-mL blood sample was collected from the right common carotid artery of each rat 6 h after LPS administration, blood samples were immediately centrifuged (2500 *g* for 10 min at 4°C) and the serum was used to measure the levels of aspartate aminotransferase (AST), alanine aminotransferase (ALT), blood urine nitrogen (BUN), and creatinine (Cr) (Fuji DRI-CHEM 3030, Fuji Photo Film, Japan). The remaining serum was immediately stored at –20°C for subsequent measurements.

### Malondialdehyde (MDA) and superoxide dismutase (SOD) activity in lung, liver, and kidney tissues

The lung, liver, and kidney tissues were removed 6 h after LPS administration, snap-frozen in liquid nitrogen and then stored at –80°C for subsequent analysis. The tissues were homogenized and centrifuged (2500 *g* for 10 min at 4°C) and the supernatant was incubated in a water bath (60°C) for 2 h for subsequent determination of MDA levels and SOD activity. The assay kits for SOD and MDA were purchased from Jianchen Bioengineering Institute (China).

### Plasma cytokines and NO concentration

A 2-mL blood sample was collected from the right common carotid artery of each rat 6 h after LPS administration. After 30 min, the blood samples were centrifuged (2500 *g* for 10 min at 4°C) and the serum samples were stored at -20°C for subsequent analysis. The serum levels of TNF-α, IL-1β, IL-6, IL-8, and IL-10 were analyzed using enzyme-linked immunosorbent assay kits (R&D Systems, USA), according to the manufacturer's instructions. The nitric oxide (NO) concentration in tissue was determined by nitrate reductase according to the manufacturer's instructions.

### Histological examination

Samples of lung, kidney, and liver tissues were taken 6 h after LPS administration. The specimens were routinely fixed and embedded in paraffin. Sections were stained with hematoxylin and eosin (H&E). A semiquantitative scoring system was adopted to evaluate the lung, liver, and kidney injury in a blind manner using light microscopy.

The histological lung injury was scored based on the alveolar congestion, hemorrhage, neutrophil infiltration into the airspace or vessel wall, and thickness of the alveolar wall (12). The histological liver injury was scored based on hepatocellular necrosis, bleeding, and inflammatory cell infiltration in the liver (12). The histological kidney injury was scored based on the inflammatory cell infiltration and glomerular capillary congestion (12). Lung, kidney, and liver sections were scored as 1: no pathological changes or very slight; 2: slight pathological changes; 3: moderate pathological changes; 4: severe pathological changes. Evaluation scores were added for the total lung, kidney, and liver renal injury score.

### Western blot analysis

The appropriate amount of lung, liver, and kidney tissue (50 mg) was taken separately, the protein lysis liquid was added, the samples were homogenized on ice, and centrifuged at 12,000 *g* for 20 min at 4°C, and supernatants collected. The protein concentration was determined by a bicinchoninic acid protein assay. Samples with equal quantities of protein were mixed with 5×SDS sample buffer and then boiled for 15 min. Aliquots of the samples were separated by 10% SDS-PAGE and electrophoresis. Following transfer to polyvinylidene fluoride (PVDF) membranes, the samples were blocked with TBS + 20% Tween 20 (TBST) solution containing 5% skimmed milk powder for 1.5 h at room temperature. The membranes were washed 3 times with TBST at room temperature, 10 min each time, and then incubated overnight at 4°C with HO-1 primary antibody (1:1000 dilution, Abcam, USA) and an anti-β-actin antibody (1:1000 dilution, Santa Cruz Biotechnology, USA). The membranes were washed 3 times with TBST at room temperature, 10 min each time, and then incubated with an HRP-conjugated secondary antibody for 2 h at room temperature. Membranes were washed 3 times with TBST at room temperature, 10 min each time, then reacted with an enhanced chemiluminescence substrate (Pierce, USA), and finally exposed to photographic film for a suitable length of time. The images were analyzed using ImageJ software (NIH, USA), and the ratios of HO-1 provided a measurement of the HO-1 levels.

### Statistical analysis

Data are reported as means±SD. The significance of differences among the four groups was tested using analysis of variance (ANOVA) for normal distribution data. Multiple comparisons were subjected to Dunnett's test. Survival analysis was done using the Kaplan-Meier method, and comparisons between groups were made using the log-rank test. All statistical analyses were performed using SPSS 11.0 for Windows (SPSS Inc., USA). Two-sided P-values <0.05 were considered statistically significant.

## Results

### Effects of DEX on the hemodynamics of rats with hemorrhage/resuscitation and endotoxemia

**Figure 1:**
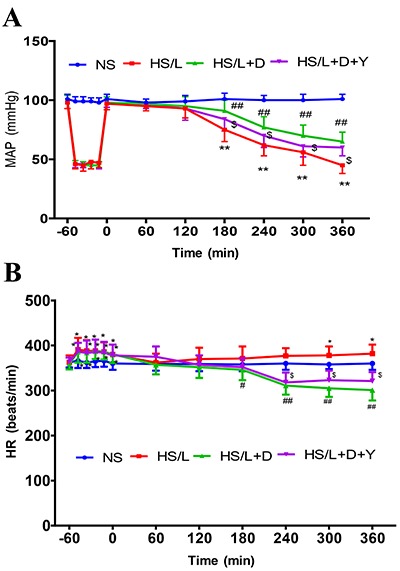
Changes in mean arterial pressure (MAP) (**A**) and heart rate (HR) (**B**) from the NS control (normal saline), hemorrhage/resuscitation plus lipopolysaccharide (HS/L), HS/L plus dexmedetomidine (HS/L+D), and HS/L+D plus yohimbine (HS/L+D+Y) groups. Data are reported as means±SD. **P<0.01, *P<0.05 *vs* the NS group; ^##^P<0.01, ^#^P<0.05 *vs* the HS/L group; ^$^P<0.05 *vs* the HS/L+D group (ANOVA). n=10/group.

There were no significant differences among the groups regarding animal weight, blood loss, resuscitation volume, and the dose of pentobarbital (all P>0.05) (Table 1). Values of MAP in the NS group remained stable throughout the experiment (Figure 1A and B). Baseline values for MAP in the NS, HS/L, HS/L+D, and HS/L+D+Y groups were similar (all P>0.05), and values for MAP 1 to 2 h after resuscitation in the NS, HS/L, HS/L+D, and HS/L+D+Y groups were also similar (all P>0.05). However, values for MAP measured from 3 to 6 h after resuscitation in the HS/L, HS/L+D, and HS/L+D+Y groups were significantly lower than those in the N/S group (all P<0.01). Values for MAP in the HS/L+D group were significantly higher than those in the HS/L group from 3 to 6 h after resuscitation (all P<0.01), and MAP values in the HS/L+D+Y group were higher than those in the HS/L+D group (all P<0.05) (Figure 1A).

**Table 1:** Baseline characteristics.

Group	Weight (g)	Blood loss volume (mL)	Resuscitation volume (mL)	Dose of pentobarbital (mg)
NS	208.40±4.09	0	0	14.17±0.28
HS/L	207.60±4.01	5.19±0.10	10.26±0.10	14.12±0.27
HS/L+D	209.10±5.13	5.23±0.13	10.41±0.25	14.22±0.35
HS/L+D+Y	205.30±4.06	5.18±0.11	10.25±0.18	13.98±0.26

Data are reported as means±SD (n=10). There were no significant differences among the groups regarding animal weight, blood loss, resuscitation volume, and the dose of pentobarbital (all P>0.05, ANOVA). NS: normal saline; HS/L: hemorrhage/resuscitation plus lipopolysaccharide; D: dexmedetomidine; Y: yohimbine.

Baseline values for HR in the NS, HS/L, HS/L+D, and HS/L+D+Y groups were similar (all P>0.05). HR was measured every 12 min from –60 to 0 min and every 1 h after resuscitation. HR values measured from -60 to 0 min in the HS/L, HS/L+D, and HS/L+D+Y groups were higher than those in the NS group (P<0.05). HR values measured from 5 to 6 h in the HS/L group were significantly higher than those in the NS group (P<0.05). Values for HR in the HS/L+D group were significantly lower than those in the HS/L group from 3 to 6 h after resuscitation (P<0.05, P<0.01). Values for HR in the HS/L+D+Y group were higher than those in the HS/L+D group from 4 to 6 h after resuscitation (all P<0.05) (Figure 1B).

### Effects of DEX on organ dysfunction caused by hemorrhage/resuscitation plus endotoxemia in rats

**Figure 2:**
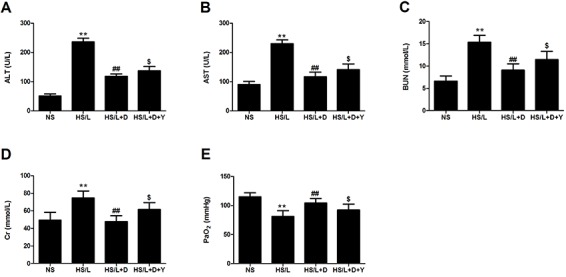
Plasma levels of (**A**) alanine aminotransferase (ALT), (**B**) aspartate aminotransferase (AST), (**C**) blood urine nitrogen (BUN), (**D**) creatinine (Cr), and (**E**) blood gas (PaO_2_) in the NS control (normal saline), hemorrhage/resuscitation plus lipopolysaccharide (HS/L), HS/L plus dexmedetomidine (HS/L+D), and HS/L+D plus yohimbine (HS/L+D+Y) groups. Data are reported as means±SD. **P<0.01 *vs* the NS group; ^##^P<0.01 *vs* the HS/L group; ^$^P<0.05 *vs* the HS/L+D group (ANOVA). n=10/group.

Biochemical assays revealed that HS plus LPS administration resulted in increased serum levels of ALT, AST, BUN, and Cr (all P<0.01) (Figure 2A to 2D). However, treatment of HS/L rats with DEX attenuated all of these increases. In addition, the levels of ALT, AST, BUN, and Cr in the HS/L+D+Y group were higher than those in the HS/L+D group (all P<0.05). Arterial blood gas data indicated that rats in the HS/L group had decreased PaO_2_ (lung function) levels (P<0.01), which were increased by DEX administration, but PaO_2_ levels in the HS/L+D+Y group were lower than those in the HS/L+D group (P<0.05).

### Effects of DEX on MDA levels and SOD activity induced by hemorrhage/resuscitation plus endotoxemia in rats

**Figure 3:**
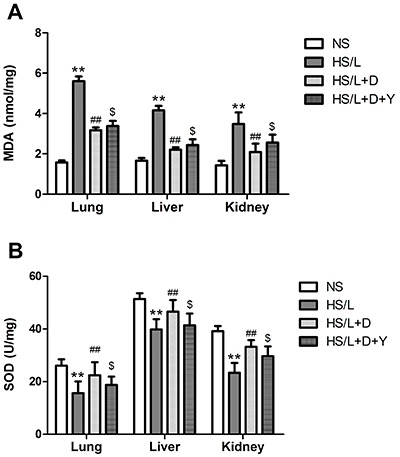
Malondialdehyde (MDA) levels (**A**) and superoxide dismutase (SOD) activity (**B**) in lung, liver, and kidney tissues of the NS control (normal saline), hemorrhage/resuscitation plus lipopolysaccharide (HS/L), HS/L plus dexmedetomidine (HS/L+D), and HS/L+D plus yohimbine (HS/L+D+Y) groups. Data are reported as means±SD. **P<0.01 *vs* the NS group; ^##^P<0.01 *vs* the HS/L group; ^$^P<0.05 *vs* the HS/L+D group (ANOVA). n=10/group.

Compared with the NS group, rats in the HS/L group had significantly increased MDA levels and reduced SOD activity in lung, liver, and kidney tissues (all P<0.01), while rats in the HS/L+D group showed significantly decreased MDA levels and increased SOD activity in those tissues compared with the HS/L group (all P<0.01). The increases in MDA levels and reductions in SOD activity in lung, liver, and kidney tissue were partially reversed by administration of yohimbine (all P<0.05) (Figure 3A and B).

### Effects of DEX on plasma levels of NO and the proinflammatory and anti-inflammatory cytokines induced by hemorrhage/resuscitation plus endotoxemia in rats

**Figure 4:**
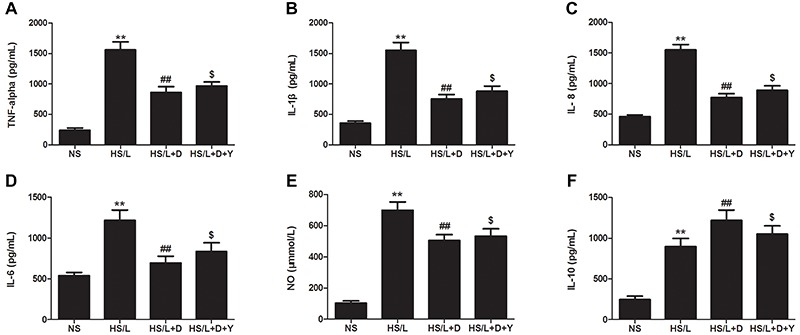
Plasma levels of TNF-α (**A**), IL-1β (**B**), IL-8 (**C**), IL-6 (**D**), NO (**E**) and IL-10 (**F**) of the NS control (normal saline), hemorrhage/resuscitation plus lipopolysaccharide (HS/L), HS/L plus dexmedetomidine (HS/L+D), and HS/L+D plus yohimbine (HS/L+D+Y) groups. Data are reported as means±SD. **P<0.01 *vs* the NS group; ^##^P<0.01 *vs* the HS/L group; ^$^P<0.05 *vs* the HS/L+D group (ANOVA). n=10/group.

Compared with the NS group, rats in the HS/L group had significantly increased plasma levels of TNF-α, IL-β, IL-6, IL-8, and NO (all P<0.01, Figure 4A-E). Compared with the HS/L group, rats in the HS/L+D group had significantly decreased plasma levels of TNF-α, IL-β, IL-6, IL-8, and NO (all P<0.01), while these increases were partially reversed in the HS/L+D+Y group (P<0.05). Compared with the NS group, rats in the HS/L group had significantly increased plasma levels of IL-10 (P<0.01). Compared with the HS/L group, rats in the HS/L+D group had further increased plasma levels of IL-10 (Figure 4F) (P<0.01), while these increases were partially reversed in the HS/L+D+Y group (P<0.05).

### Effects of DEX on the histopathology of lung, liver, and kidney caused by hemorrhage/resuscitation plus endotoxemia in rats

**Figure 5:**
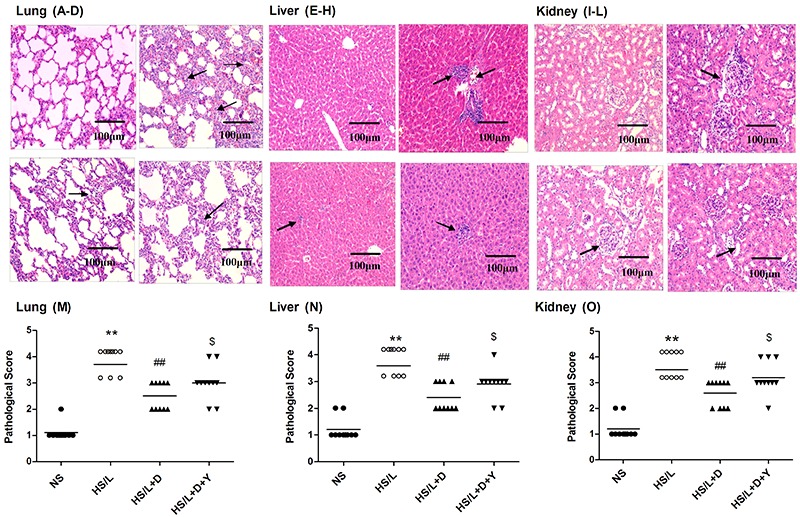
Representative microscopic findings of the lung (**A**-**D**), liver (**E**-**H**), and kidney (**I**-**L**) tissues of the NS control (normal saline, n=10), hemorrhage/resuscitation plus lipopolysaccharide (HS/L, n=10), HS/L plus dexmedetomidine (HS/L+D, n=10), and HS/L+D plus yohimbine (HS/L+D+Y, n=10) groups (hematoxylin and eosin;×200, magnification bar: 100 μm). Arrows indicate pathological changes. Histological injury scores for lung (**M**), liver (**N**), and kidney (**O**) tissues. Data are reported as means±SD. **P<0.01 *vs* the NS group; ^##^P<0.01 *vs* the HS/L group; ^$^P<0.05 *vs* the HS/L+D group (ANOVA). n=10/group.

Histological analyses revealed normal lung, liver, and kidney tissues in the NS group (Figure 5A, E, I); severe lung, liver, and kidney injuries in the HS/L group (Figure 5B, F, J); and only mild-to-moderate lung, liver, and kidney injuries in the HS/L+D (Figure 5C, G, K) and HS/L+D+Y groups (Figure 5D, H, L). Compared with the NS group, rats in the HS/L group showed higher lung, liver, and kidney injury scores (all P<0.01). Compared with the HS/L group, rats in the HS/L+D group showed lower lung, liver, and kidney (Figure 5 M–O) injury scores (all P<0.01), while these increases were partially reversed in the HS/L+D+Y group (P<0.05).

### Effects of DEX on HO-1 protein expression of lung, liver, and kidney caused by hemorrhage/resuscitation plus endotoxemia in rats

**Figure 6:**
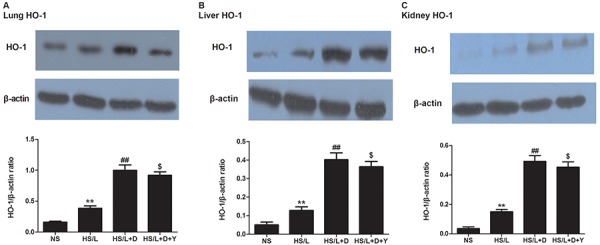
Heme oxygenase-1 (HO-1) protein expression in the lung (**A**), liver (**B**), and kidney (**C**) of the NS control (normal saline), hemorrhage/resuscitation plus lipopolysaccharide (HS/L), HS/L plus dexmedetomidine (HS/L+D), and HS/L+D plus yohimbine (HS/L+D+Y) groups. Data are reported as means±SD. **P<0.01 *vs* the NS group; ^##^P<0.01 *vs* the HS/L group; ^$^P<0.05 *vs* the HS/L+ D group (ANOVA). n=10/group.

Compared with the NS group, rats in the HS/L group had increased HO-1 protein levels in lung, liver, and kidney tissue (P<0.01). Compared with the HS/L group, rats in the HS/L+D group had significantly increased HO-1 protein levels in lung, liver, and kidney tissue (Figure 6A-C) (P<0.01), while these increases were partially reversed in the HS/L+D+Y group (P<0.05).

### Effects of DEX on the survival rate of rats treated with hemorrhage/resuscitation plus endotoxemia

**Figure 7:**
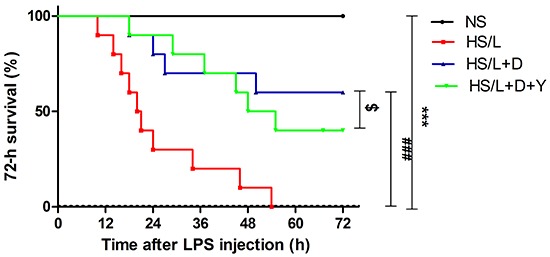
Comparison of 72-h survival of rats treated with normal saline (NS), hemorrhage/resuscitation plus lipopolysaccharide (HS/L), HS/L plus dexmedetomidine (HS/L+D), and HS/L+D plus yohimbine (HS/L+D+Y). ***P<0.001 *vs* NS group; ^###^P<0.001 *vs* HS/L group; ^$^P<0.05 *vs* HS/L+D group (ANOVA). n=10/group.

No rats died in the NS group, but all rats in the HS/L group died within 54 h after LPS injection. Compared with the HS/L group, rats in the HS/L+D group had an increased survival rate within 72 h (P<0.01), while these increases were partially reversed in the HS/L+D+Y group (P<0.05) (Figure 7).

## Discussion

Previous studies were mainly limited to the protective effect of dexmedetomidine on a single organ induced by a model (7–9), but the clinical reality is more complicated. Hemorrhage/resuscitation and subsequent endotoxemia are common in clinical practice, and multiple organ damage induced by this two-hit model is more in line with clinical practice. Therefore, we chose a hemorrhage/resuscitation and subsequent endotoxemia two-hit model to observe whether dexmedetomidine had a protective effect on multiple organs such as the lung, liver, and kidney. Our study showed for the first time that DEX can attenuate lung injuries, as well as renal and liver dysfunction, in a two-hit model of hemorrhage/resuscitation and endotoxemia in rats. Our results also showed that DEX improved survival in rats with HS/L. In addition, the multiorgan protective effects of DEX were partly reversed by yohimbine, an antagonist of the α_2_-adrenergic receptors. DEX has been reported to provide effective sedative effects to critically ill patients (13). The findings of the present study could be beneficial to patients with hemorrhage/resuscitation and subsequent endotoxemia.

The data also revealed that DEX reduced inflammation resulting from damaged lung, liver, and kidney tissue, and serum released after hemorrhage/resuscitation and subsequent endotoxemia, thereby increasing the survival of HS/L-treated rats. These data seem to support the concept that DEX might be beneficial in clinical situations similar to those represented in the HS/L group of rats. However, previous studies have administered DEX at doses higher than a clinical dose to show that it effectively attenuated an inflammatory response or organ injury (14). Actual clinical trials are required to verify the ability of DEX to protect organs in this clinical context.

The increased release of proinflammatory cytokines plays an important role in the initiation and perpetuation of organ injury (15). Remick et al. (16) reported that IL-6 levels 6 h after induction of sepsis were predictive of mortality in a rodent model of sepsis. Furthermore, Kraft et al. (17) demonstrated that elevated levels of IL-8 correlated with increased rates of MOD, sepsis, and mortality. Liu et al. (18) showed that increased levels of TNF-α and TNF receptors (TNFRs) were associated with the severity of trauma-provoked organ dysfunction. These combined data suggest that serum levels of proinflammatory cytokines may be a valid biomarker for monitoring organ dysfunction. Previous studies revealed that DEX decreased LPS-induced levels of TNF-α, IL-1, IL-6, and IL-8 production in both murine macrophages (19) and human whole blood *in vitro* (20). When proinflammatory cytokines are released, anti-inflammatory cytokines are also released. In a previous study, IL-10 inhibited the release of TNF-α in mice induced by LPS and reduced the mortality of a lethal dose of LPS (21). Wu et al. reported that IL-10 therapy could inhibit the activation of neutrophils and the secretion of proinflammatory cytokines, which is protective in LPS-induced ALI (22). In the present study, increased plasma levels of TNF-α, IL-β, IL-6, and IL-8 induced by HS/L could be attenuated by DEX treatment. The results also showed that hemorrhage/resuscitation induced an increase in IL-10 levels and that DEX treatment further increased IL-10 levels, indicating that the protective properties of DEX against organ dysfunction are associated with reduced proinflammatory and increased anti-inflammatory processes.

Oxidative stress has been shown to play an important role in organ failure (23). SOD exerts strong antioxidant effects. MDA is the end-product of lipid peroxidation. In this study, the levels of MDA and SOD were assessed in lung, liver, and kidney tissues to evaluate the effects of DEX on oxidative stress. The results suggested that organ injuries in the HS/L rats resulted in increased MDA levels and decreased SOD activity, which could be reversed by DEX. DEX exerts potent antioxidant effects (24). Furthermore, DEX has been shown to decrease MDA levels and increase SOD activity in patients with lung cancer receiving one-lung ventilation (25). These findings suggest that the mechanisms underlying the therapeutic effects of DEX in humans may be similar to those that allow it to attenuate oxidative stress in HS/L-treated rats.

The hemodynamic data of the present study indicated that HS/L decreased MAP and increased HR in rats, while administration of DEX increased MAP and reduced HR. Thus, it is likely that the mechanism by which DEX alleviates hemodynamic alterations may also contribute to its therapeutic effects. A previous study reported that DEX increased vascular reactivity, reduced vasopressor requirements in cases of septic shock, and increased blood pressure (26). Another study reported that DEX increased the pressor response to norepinephrine without adverse consequences on tissue perfusion in rats with sepsis. (27,28) Although the alfa-2 adrenergic receptor is a known mechanism of controlling blood pressure by negative feedback on sympathetic neuronal fibers, this inhibitory effect of dexmedetomidine has a small effect on blood pressure (11,29). In addition, inflammatory cytokines such as TNF-α, IL-β, and NO can inhibit the systolic function of the heart and dilating peripheral blood vessels (30–32), and these inflammatory cytokines can damage the capillary endothelial cells, causing capillary leakage, thereby reducing the effective circulation capacity (33). Therefore, the above factors are the main causes of hypotension induced by hemorrhage/resuscitation and subsequent endotoxemia. In this study, we observed that DEX inhibited the release of these inflammatory cytokines induced by hemorrhage/resuscitation and subsequent endotoxemia. In summary, DEX prevented a further blood pressure drop mainly by inhibiting the release of inflammatory cytokines, while the influence of alfa-2 adrenergic receptor-mediated sympathetic negative feedback was relatively small.

HO-1, also known as heat shock protein 32, is a rate-limiting enzyme that catalyzes the production of heme. Studies have shown that HO-1 plays anti-inflammatory and antioxidant roles in a mouse model of acute pancreatitis by inhibiting the NF-κB signaling pathway (34), which may be beneficial for increasing blood pressure. Although the HO-1/carbon monoxide pathway is a known vascular diastolic mechanism, carbon monoxide at the same time has been shown to play anti-inflammatory, antioxidant, and organ-protective roles (35,36). As a result, HO-1 exerts anti-inflammatory effects not only by inhibiting the NF-κB signaling pathway but also through the production of the anti-inflammatory compound carbon monoxide. Enhanced HO-1 expression after hemorrhagic shock and subsequent resuscitation in multiple organs (including the kidney, liver, and lung) might be responsible for the organ protection and inflammation inhibitory effects (37). Recently, Gao et al. (25) reported that DEX increased the levels of HO-1 expression and reduced oxidative stress during one-lung ventilation. In the present study, DEX treatment resulted in significantly higher HO-1 protein expression in major organs, including the lung, liver, and kidney. Therefore, DEX-induced increased MAP may be mainly related to the anti-inflammatory and antioxidant effects of HO-1, and the effect of HO-1/CO pathway vasodilatation on blood pressure is relatively small.

α_2_-adrenoceptors were first discovered in the pre-synapse of the adrenergic neurons in the central and peripheral nervous systems. The physiologic responses elicited by activation of α_2_-adrenergic receptors include decreased salivation and pain and increased sedation. Nevertheless, a recent study by Takahashi et al. (38) has shown that DEX inhibited glucose-stimulated insulin secretion in a concentration-dependent manner, but this effect was reversed by yohimbine, an α2-adrenoceptor blocker. Another study also reported that yohimbine could completely neutralize the protective effect of DEX on oxidative damage of alveolar macrophages induced by H_2_O_2_ (39). These studies suggest that α2-adrenoceptors may also be present in some non-neuronal cell types in humans. It was previously reported that activation of α_2_-adrenergic receptors decreased the levels of various inflammatory factors and protected against LPS-induced acute kidney injuries (8). Shen et al. (7) reported that stimulation of the α_2_-adrenergic receptors reduced pulmonary damage and inhibited sterile inflammation induced by lung I/R injuries. In the present study, administration of an α_2_-adrenergic receptor antagonist (yohimbine) reduced the effects of DEX on the inflammatory response and oxidant stress and counteracted the organ protective effects of DEX. These results suggest that DEX protected against HS/L-induced multiorgan injury by activating α_2_-adrenergic receptors. Clinically, DEX is usually used to calm patients in critical situations. Our data and previous studies showed that DEX reduces the inflammatory response and organ damage induced by hemorrhagic shock and infection. Future research should focus on the anti-inflammatory effect of α2-adrenergic receptor and whether DEX is effective at treating critically ill patients.

In summary, DEX mitigated multiple-organ injury in a two-hit rat model of hemorrhagic/resuscitation and subsequent endotoxemia by inhibiting both the inflammatory response and oxidative stress through the induction of HO-1. These effects were mediated, at least in part, by α_2_-adrenoceptors.
